# Prospective Comparison of F-18 Choline PET/CT Scan Versus Axial MRI for Detecting Bone Metastasis in Biochemically Relapsed Prostate Cancer Patients

**DOI:** 10.3390/diagnostics7040056

**Published:** 2017-10-17

**Authors:** Wouter Huysse, Frédéric Lecouvet, Paolo Castellucci, Piet Ost, Valerie Lambrecht, Carlos Artigas, Marie-Laurence Denis, Kathia De Man, Louke Delrue, Lennart Jans, Aurélie De Bruycker, Filip De Vos, Gert De Meerleer, Karel Decaestecker, Valerie Fonteyne, Bieke Lambert

**Affiliations:** 1 Department of Radiology, Ghent University Hospital, De Pintelaan 185, 9000 Ghent, Belgium; Wouter.huysse@ugent.be (W.H.); Valerie.Lambrecht@uzgent.be (V.L.); Lennart.Jans@ugent.be (L.J.); 2 Department of Radiology, Centre du Cancer and Institut de Recherche Expérimentale et Clinique, Cliniques Universitaires Saint-Luc, Université Catholique de Louvain, Avenue Hippocrate 10/2942, B-1200 Brussels, Belgium; frederic.lecouvet@uclouvain.be (F.L.); mldenis@cybernet.be (M.-L.D.); 3 Service of Nuclear Medicine, Policlinico Sant’Orsola-Malpighi, University of Bologna, 40126 Bologna, Italy; paolo.castellucci@aosp.bo.it; 4 Department of Radiation Oncology and Experimental Cancer Research, Ghent University, De Pintelaan 185, 9000 Ghent, Belgium; Piet.ost@ugent.be (P.O.); Aurelie.debruycker@ugent.be (A.D.B.); valerie.fonteyne@uzgent.be (V.F.); 5 Nuclear Medicine, Institut Jules Bordet, Waterloolaan 121, 1000 Brussels, Belgium; carlos.artigas@bordet.be; 6 Department of Nuclear Medicine, Ghent University Hospital, De Pintelaan 185, 9000 Ghent, Belgium; 7 Laboratory of Radiopharmacy, Faculty of Pharmaceutical Sciences, Ghent University, Ottergemsesteenweg 460, 9000 Ghent, Belgium; Louke.Delrue@uzgent.be; 8 Radiology and Nuclear Medicine, Ghent University, De Pintelaan 185, 9000 Ghent, Belgium; FilipX.DeVos@UGent.be (F.D.V.); Bieke.Lambert@gmail.com (B.L.); 9 Department of Radiation Oncology and Experimental Cancer Research, UZ Leuven, Herestraat 49, 3000 Leuven, Belgium; Gert.Demeerleer@ugent.be; 10 Department of Urology and Experimental Cancer Research, Ghent University Hospital, De Pintelaan 185, 9000 Ghent, Belgium; Karel.Decaestecker@ugent.be; 11 Algemeen ziekenhuis Maria Middelares, Buitenring Sint-Denijs 30, 9000 Ghent, Belgium

**Keywords:** choline PET/CT, MRI, bone metastasis, prostate cancer

## Abstract

We compared fluor-18 choline positron emission tomography/computed tomography (PET/CT) and axial skeleton magnetic resonance imaging (MRI) prospectively obtained for the detection of bone metastases in non-castrated patients with biochemically recurrent prostate cancer following primary treatment. PET/CT was performed 45 min post-injection of 3–4 MBq/kg F-18 methyl choline. MRI included T1- and fluid sensitive T2-weighted images of the spine and pelvis. Readers were initially blinded from other results and all scans underwent independent double reading. The best valuable comparator (BVC) defined the metastatic status. On the basis of the BVC, 15 out of 64 patients presented with 24 bone metastases. On a patient level, the sensitivity and specificity of MRI and PET were not significantly different. On a lesion level, the sensitivity of MRI was significantly better compared to PET, and the specificity did not differ significantly. In conclusion, axial MRI is an interesting screening tool for the detection of bone metastases because of its low probability of false negative results. However, F-18 choline PET is a valuable addition as it can overrule false positive MRI results and detect non-axial metastases.

## 1. Introduction

A proportion of patients diagnosed with a rising prostate-specific antigen (PSA) following treatment of prostate cancer with curative intent will develop a clinical recurrence during their disease course. Some patients will do so in limited volume, for which metastasis-directed therapy is an investigational approach [1]. However, traditional imaging studies, such as bone scan and computed tomography, lack sufficient sensitivity to detect low volume metastatic disease at low PSA levels [2]. Consequently, these imaging modalities are not recommended to detect metastases in asymptomatic patients until the PSA rises above 10 ng/mL or high PSA kinetics are detected. The role of functional imaging modalities such as Choline positron emission tomography/computed tomography (PET/CT) has shown promising results to detect low volume metastases at low PSA levels. The European Association of Urology (EAU) suggest referring patients for C-11 or F-18 choline PET/CT in case the PSA rises >1 ng/mL if the result is expected to impact patient management [3].

In case of patients with limited metastatic disease, metastasis-directed therapy (MDT) by means of surgery or external beam radiotherapy, is currently being investigated as a novel therapeutic strategy [1,4]. In Belgium, the Surveillance or metastasis-directed Therapy for OligoMetastatic Prostate cancer recurrence (STOMP) trial—a randomized trial comparing surveillance with metastasis directed therapy MDT—is being performed with all eligible patients being screened with F-18 choline PET/CT [5,6]. At the time of study initiation (2012), there was a lack of prospective data concerning the sensitivity and specificity of F-18 choline PET/CT for the detection of bone metastases. Previous studies have indicated that whole body magnetic resonance imaging (MRI) or axial skeleton MRI outperforms bone scintigraphy for the detection of bone metastases in both the primary as the recurrent setting [7]. Consequently, patients potentially eligible for the STOMP trial were screened with both axial skeleton MRI and F-18 choline PET/CT in order to establish their diagnostic accuracy for bone metastases.

## 2. Materials and Methods

We recruited 86 consecutive patients with a biochemical relapse [3] following local prostate cancer treatment and testosterone levels >50 ng/dL at time of inclusion. The study was approved by our local Ethics Committee (EC 2012/308) and patients signed informed consent.

PET/CT was performed 45 min following injection of 3–4 MBq/kg F-18 methyl Choline. Patients were scanned from the base of skull to the proximal thighs. All PET/CT scans were co-reported by two senior staff members of nuclear medicine and radiology (Bieke Lambert, Louke Delrue). Double reading was performed by experienced nuclear medicine specialists (Paolo Castellucci, Carlos Artigas, Kathia De Man). MRI included the entire spine with 3 mm sagittal short T1 inversion recovery (STIR) and T1-weighted images, and pelvis with 5 mm coronal T1-weighted and fat saturated proton-density and T2-weighted images. All scans were read in twofold by experienced musculoskeletal radiologists (Wouter Huysse, Frédéric Lecouvet, Marie-Laurence Denis). In case of conflicting interpretation of the scans, a third reader (VL) was appointed as adjudicator. Readers were initially blinded from other scans, patient information and biological results. Panel reviews of initial and follow-up imaging findings, with all available baseline and follow-up clinical and biologic data, were used as the best valuable comparator (BVC) to define the true metastatic status [8]. A lesion-based and patient-based analysis was conducted. We excluded patients in whom the interval between both imaging studies exceeded 6 weeks. We also excluded patients who received systemic treatment or without follow-up imaging making a formal evaluation of the metastatic status impossible. Therefore, 22 patients out of 86 recruited patients were excluded for the current analysis. Patient characteristics can be found in the Table S1. The sensitivity and specificity of these approaches were compared using the McNemar test, with *p* < 0.05 considered statistically significant.

## 3. Results

In 15 out of 64 patients, 24 bone metastases were detected. Seven patients had a single lesion, four patients presented with two lesions and three lesions were found in two patients. Two additional patients suffered proven diffuse (>5 lesions) involvement, which could not be numerically correctly accounted for in the lesion based analysis and were therefore considered as a single positive reading.

**Figure 1:**
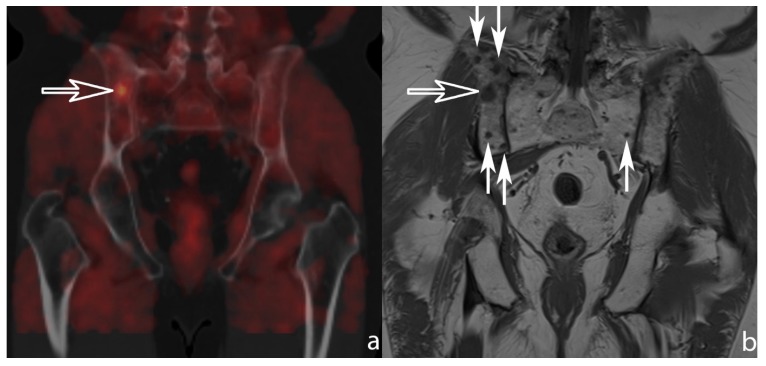
(**a**) Coronally reformatted fused choline positron emission tomography/computed tomography (PET/CT) image (left) and (**b**) 5 mm coronal T1-weighted magnetic resonance (MR) image of the sacro-iliac joints. In this patient three suspected bone metastases were reported on the PET and CT images making this patient eligible for metastasis-directed therapy. One of the lesions was located posteriorly in the right iliac bone (open arrow). On MRI, however, multiple T1-hypo-intense nodules (small arrows) can be observed indicative of diffuse metastatic disease warranting systemic treatment.

The sensitivity, specificity, positive predictive value (PPV) and negative predictive value (NPV) of MRI and PET on both patient and lesion level are depicted in Table 1. On a patient level, the sensitivity and specificity were not significantly different between MRI and PET (*p* = 0.5 and 0.5, respectively). On a lesion level, the sensitivity of MRI (Figure 1) was significantly better compared to PET (*p* = 0.031). The specificity was not significantly different between MRI and PET (*p* = 0.125).

If CT observations had been considered in combination with PET, only a single patient would have been incorrectly categorized as negative for bone metastases (sensitivity 93%, 95% CI: 0.66–0.99). Regarding the lesion-based level, the combination with CT correctly assigned 21/24 lesions, resulting in a sensitivity of 88% (95% CI 0.67–0.97). However, since two patients with diffuse bone involvement were heavily underestimated on both PET and CT, which we cannot numerically account for, the calculated sensitivities for F18 choline PET/CT are probably overrated. 

## 4. Discussion

We addressed axial MRI versus F-18 choline PET/CT on a patient level as well as in a per-lesion assessment. Non-castrated patients with a biochemical relapse following radical treatment of their prostate cancer represent a heterogeneous population. Various research groups reported that patients with low volume disease have a different tumor biology and prognosis compared to patients presenting with high volume disease (“polymetastic disease”, >3 lesions) [6,9,10,11]. In the ongoing STOMP trial, we recruit patients with low volume disease recurrence (up to three metastatic lesions, so-called “oligometastatic disease”). They are offered metastasis directed treatment, e.g., surgery or external beam radiotherapy. In our analysis, PET/CT with F-18 choline underestimated the polymetastatic nature of the disease in two patients.

However, most patients do not suffer relapse disease limited to the axial skeleton [6]. In a large prospective cohort (208 patients) from the Ghent University Hospital, three out of four patients with a biochemical relapse following radical treatment for prostate cancer were categorized as low volume disease, and in only 18% it concerned bone-only disease. The majority of patients presented with lymph node involvement or a combination of nodal and skeletal metastasis. Therefore, the whole body approach obtained by PET/CT is considered complementary to the axial MRI.

The advantages of both imaging modalities are available in hybrid PET/MRI scanners showing promising data for relapsed prostate cancer [12], with an improved detection rate for bone metastasis due to the MR component and a better evaluation of lymph nodes due to the use of PET. The recent introduction of gallium-68 PSMA (prostate specific membrane antigen) as a PET tracer might further improve results.

Recently, gallium-68 labeled prostate specific membrane antigen (Ga-68 PSMA) was introduced as a PET/CT-tracer for restaging low volume prostate cancer. The yield of positive scans, in particular at very low PSA levels, was proven to be higher for Ga-68 PSMA than obtained with radiolabeled choline [13,14]. Non-controlled studies comparing Choline and PSMA also suggest a higher yield for bone metastasis using PSMA [15]. However, validation by means of pathology or clinical follow-up is still awaited in order to determine the sensitivity and specificity of Ga-68 PSMA in this patient population.

## 5. Conclusions

In conclusion, both axial MRI and F-18 choline PET are valuable screening tools for the detection of bone metastasis in prostate cancer. MRI outperformed choline PET/CT in terms of sensitivity in a lesion-based analysis. However, the high specificity of choline PET/CT proved useful to overrule false positive MRI results. Moreover, choline PET/CT allows for the detection of non-axial bone metastases and metastatic lymph nodes.

## Figures and Tables

**Table 1:** Imaging results for predicting bone metastases on best value comparator.

Patient-Based Analysis								
**BVC for bone metastases**								
Image modality	Scan result	**Negative (*n* = 49)** **No.**	**Positive (*n* = 15)** **No.**	**Total (*n* = 64)** **No.**	**Sensitivity % (95% CI)**	**Specificity % (95% CI)**	**PPV % (95% CI)**	**NPV % (95% CI)**
Ax MRI	Negative	47	0	47	100 (75–100)	96 (84–99)	88 (62–98)	100 (91–100)
	Positive	2	15	17				
F-18 Choline PET	Negative	49	2	51	87 (58–98)	100 (91–100)	100 (72–100)	96 (85–99)
	Positive	0	13	13	*p* = 0.5	*p* = 0.5		
**Lesion-based analysis**								
Image modality	Scan result	**Negative (*n* = 55)** **No.**	**Positive (*n* = 24)** **No.**	**Total (*n* = 79)** **No.**	**Sensitivity % (95% CI)**	**Specificity % (95% CI)**	**PPV % (95% CI)**	**NPV % (95% CI)**
Ax MRI	Negative	51	0	51	100 (83–100)	93 (82–98)	86 (66–95)	100 (91–100)
	Positive	4	24	28				
F-18 Choline PET	Negative	55	6	61	75 (53–89)	100 (91–100)	100 (78–100)	90 (79–96)
	Positive	0	18	18	*p* = 0.031	*p* = 0.125		

BVC = best value comparator, PPV = positive predictive value, NPV = negative predictive value, CI = confidence interval, Ax MRI = axial skeleton magnetic resonance imaging, PET/CT = positron emission tomography/computed tomography.

## Supplementary Materials

Supplementary materials can be found at www.mdpi.com/2075-4418/07/4/56/s1.
