# Supplementary material for: Prospective Comparison of F-18 Choline PET/CT Scan Versus Axial MRI for Detecting Bone Metastasis in Biochemically Relapsed Prostate Cancer Patients
Source: Diagnostics (Basel). 2017 Oct 17;7(4):56. doi: 10.3390/diagnostics7040056 (PMC5745392; doi:10.3390/diagnostics7040056)
Supplement: Supplementary file 1 [file diagnostics-07-00056-s001.pdf]

Table S1. patient characteristics.

Characteristic	All Patients (*n* = 64)
**Primary treatment modality at PCa diagnosis**	
Radical prostatectomy with or without postoperative RT	46 (71.9%)
Radiotherapy with or without adjuvant ADT	18 (28.1%)
**PSA at PCa diagnosis (ng/ml)**	
Median (IQR)	12.0 (7.5–16.9)
Unknown	9
**EAU prognostic grouping at PCa diagnosis**	
Localized	22 (34%)
Locally advanced	44 (66%)
**Interval from PCa diagnosis to imaging (yr)**	
Median (IQR)	4.7 (3.6–7.3)
**PSA level at time of imaging (ng/ml)**	
Median (IQR)	3.1 (1.2–6.5)
**PSA DT at time of imaging (mo)**	
Median (IQR)	5.9 (3.39.8)
Unknown	2

* Abbreviations: yr = year, mo = months, IQR = interquartile range, EAU = European Association of Urology, PCa = prostate cancer, PSA-DT = prostate specific
